# Chemical synthesis of hierarchical NiCo_2_S_4_ nanosheets like nanostructure on flexible foil for a high performance supercapacitor

**DOI:** 10.1038/s41598-017-10218-z

**Published:** 2017-08-29

**Authors:** D. -Y. Kim, G. S. Ghodake, N. C. Maile, A. A. Kadam, Dae Sung Lee, V. J. Fulari, S. K. Shinde

**Affiliations:** 1 Department of Biological and Environmental Science, College of Life Science and Biotechnology, Dongguk University-Seoul, Biomedical Campus-Ilsan, Goyang-si, Gyeonggi-do, 10326 South Korea; 2 Holography and Materials Research Laboratory, Department of Physics, Shivaji University, Kolhapur, 416 004 Maharashtra India; 3 Research Institute of Biotechnology and Medical Converged Science, Dongguk University, Biomedi Campus, Ilsandong-gu, Goyang-si, Gyeonggi-do, 10326 Republic of Korea; 4 Department of Environmental Engineering, Kyungpook National University, 80 Daehak-ro, Buk-Gu, Daegu, 41566 Republic of Korea

## Abstract

In this study, hierarchical interconnected nickel cobalt sulfide (NiCo_2_S_4_) nanosheets were effectively deposited on a flexible stainless steel foil by the chemical bath deposition method (CBD) for high-performance supercapacitor applications. The resulting NiCo_2_S_4_ sample was characterized by X-ray powder diffraction (XRD), field emission scanning electron microscopy (FE-SEM), high-resolution transmission electron microscopy (HR-TEM), and electrochemical measurements. XRD and X-ray photoelectron spectroscopy (XPS) results confirmed the formation of the ternary NiCo_2_S_4_ sample with a pure cubic phase. FE-SEM and HR-TEM revealed that the entire foil surface was fully covered with the interconnected nanosheets like surface morphology. The NiCo_2_S_4_ nanosheets demonstrated impressive electrochemical characteristics with a specific capacitance of 1155 F g^−1^ at 10 mV s^−1^ and superior cycling stability (95% capacity after 2000 cycles). These electrochemical characteristics could be attributed to the higher active area and higher conductivity of the sample. The results demonstrated that the interconnected NiCo_2_S_4_ nanosheets are promising as electrodes for supercapacitor and energy storage applications.

## Introduction

In recent years, supercapacitor/energy storage devices have emerged as devices with great potential because of the rapid expansion of new and environmentally friendly energy conversion and storage devices^1–7^. Supercapacitors, which are useful energy storage devices for hybrid electric vehicles, and batteries, have attracted considerable attention because of their high power density and long-life cycling stability that are comparable to other batteries^4, 8–11^. The capabilities of supercapacitors mostly depend on the active electrode materials, and they can generally be divided into three main types: transition metal oxides, carbon materials, and conducting polymers^4^.

Various transition metal oxides are used in supercapacitors, such as RuO_2_^12^, NiO^13^, ZnO/NiO^14^, CuO^15^, NiCoO_2_^16^, CuO^17^, CoO^18, 19^, FeO_2_^20^, and MnO_2_^21^, and metal sulfides such as NiS^22^, MnS^23, 24^, CoS^25^, CoS_2_^26^, and CuCo_2_O_4_;^27^ these are attractive electrode materials for supercapacitor applications. Recently, transition metal chalcogenides are increasingly used in supercapacitor applications. Sulfur ions tend to produce flexible nanostructures because of higher electroconductive properties and fast charge transportation^28^. In comparison with binary and ternary metal oxides, ternary metal sulfides are more popular because of the higher levels active redox species, fast charge-discharge, and long-time stability. Among them, nickel cobalt sulfide (NiCo_2_S_4_) has attracted significant interest because of its environmentally stable nature, high redox reactions^29^, high theoretical specific capacitance, and high electronic conductivity^30^.

To date, thin films of ternary NiCo_2_S_4_ have been widely studied by various researchers. The films have been deposited using various techniques such as the calcination^31^, hydrothermal^32^ via a gas bubble soft template and hydrothermal^33^, the sputtering^34^, and the sulfur-bubble template methods^35^. Among the various available methods, the chemical bath deposition method is one of the simple, cheap, and attractive method.

Yu *et al*.^36^. have prepared NiCo_2_S_4_ using the hydrothermal method. They reported that the capacitance was 720 mAh g^−1^ after 50 cycles, which was similar to the theoretical capacity of NiCo_2_S_4_ electrodes. Jia *et al* ^37^. have synthesized NiCo_2_S_4_ electrodes and NiCo_2_S_4_@Fe_2_O_3_ on Ti substrates using a simple electrodeposition method for asymmetric supercapacitor application. The research group developed an asymmetric supercapacitor with the NiCo_2_S_4_/Fe_2_O_3_ electrode. The specific capacitance of the asymmetric cell was determined as 342 F g^−1^ at a scan rate of 5 mV s^−1^. In another study, Li *et al* ^38^. described a facile and commendable method to produce hierarchical NiCo_2_S_4_/Co(OH)_2_ nanotubes on Ni foam. They reported that NiCo_2_S_4_/Co(OH)_2_ electrodes have a high area capacitance compared with NiCo_2_S_4_ electrodes. Zhu *et al* ^39^. have prepared NiCo_2_S_4_ thin films via the solvothermal route for supercapacitor applications. They developed NiCo_2_S_4_ nanoparticles with an ultrahigh specific capacitance of 1440 F g^−1^ at 3 A g^−1^ after 250 cycles. Su *et al* ^40^. have synthesized NiCo_2_S_4_ electrodes using the solvothermal method for dye-sensitized solar cell (DSC) application, demonstrated the maximum cell efficiency of 8.94% on an ITO-coated glass substrate.

By using a facile chemical bath deposition method in this study, we effectively synthesized interconnected NiCo_2_S_4_ nanosheets on a flexible stainless steel foil for high-performance supercapacitor applications. The specific capacitance of the as-synthesized NiCo_2_S_4_ nanosheets revealed good cycling stability and a long charging-discharging time. Our results indicated that the interconnected NiCo_2_S_4_ nanosheets can be used as high-performance materials for supercapacitor applications.

## Experimental Section

### Materials

0.1 M nickel (II) sulfate (NiSO_4_(H_2_O)_6_), 0.2 M cobalt (II) sulfate (CoSO_4_∙7H_2_O), and 0.2 M sodium sulfide (Na_2_S 6H_2_O) were dissolved in 20 mL of deionized water with ammonia (NH_3_).

### Synthesis and growth mechanism of NiCo_2_S_4_

**Figure 1:**
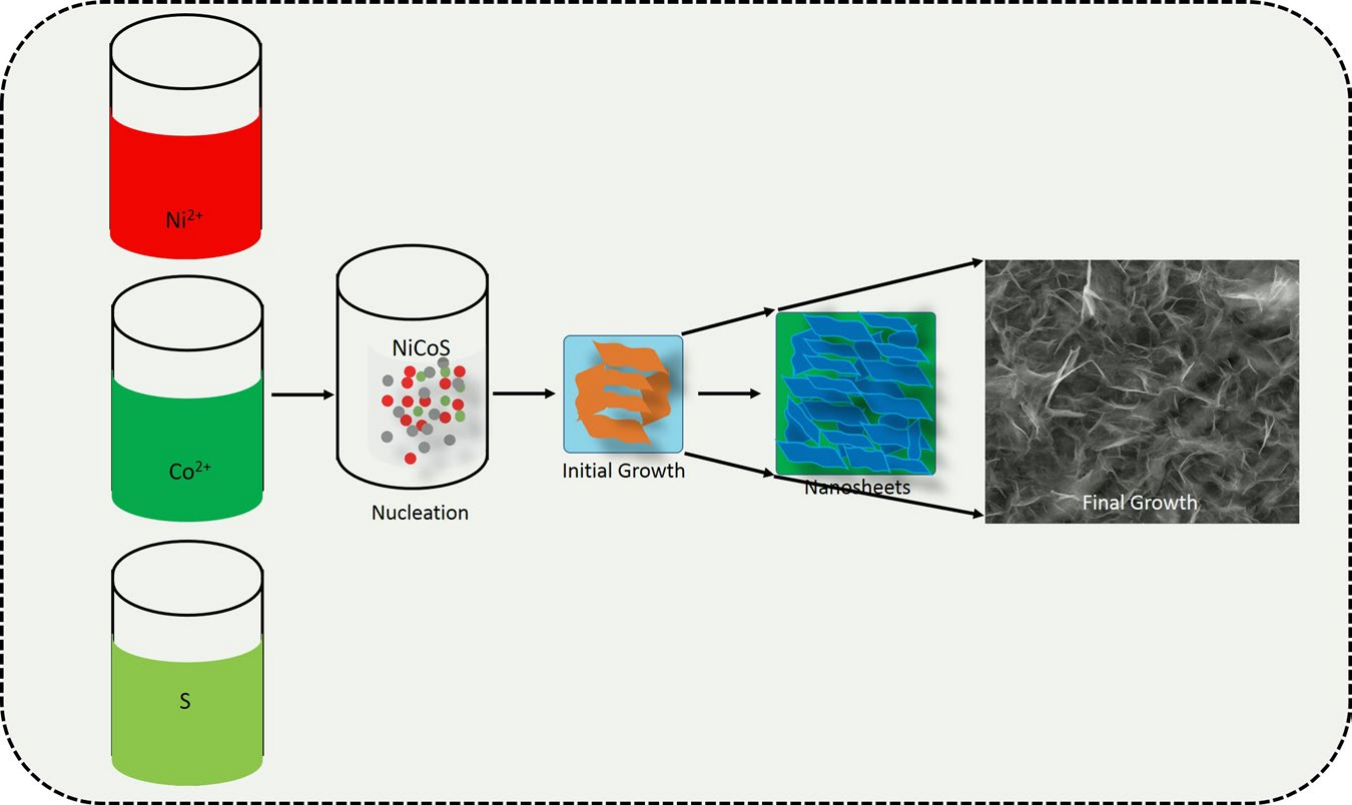
Schematic illustration of the preparation process of the nanosheets like NiCo_2_S_4_ thin films.

The proposed growth mechanism of the chemical bath deposited NiCo_2_S_4_ nanosheet-like nanostructured thin films is explained using following steps. Initially, to generate Ni^2+^ and Co^2+^, the nickel(II) sulfate (NiSO_4_(HO)_6_) and cobalt(II) sulfate (CoSO_4_∙7H_2_O) were dissolved in the double-distilled water, sodium sulfide (Na_2_S∙6H_2_O) was used as the precipitant for S^2−^ ions, and ammonia was used as a complexing agent for adjusting the pH to 11. Then, a well cleaned flexible stainless steel foil was immersed in the prepared bath and maintained at room temperature. During precipitation, nickel cobalt sulfide was deposited on the foil. After 2 h, the flexible nickel cobalt sulfide thin films deposited on the stainless steel foil was removed from the solution bath, washed with double-distilled water, dried in ambient air, and preserved in an airtight container. Deposition time is associated with nucleation ratio and growth activities. The attached nanoparticle developed along a specific crystal orientation according to the attachment and arrangement of the 3D nanostructure as shown in Fig. 1. The developmental steps of the 3D interconnected nanosheets of nickel cobalt sulfide were as follows: nucleation, growth, and oriented attachment of the nanomaterial. Nucleation is related to the total volume of the supersaturation ions in the solution. In the nucleation stage, supersaturation is extremely high, and electrostatic repulsive barriers are low; hence, particles tend to aggregate^41^.

### Characterization

The structures and morphology of NiCo_2_S_4_ thin films were characterized by X-ray powder diffraction (XRD; CuK_α_ radiation, λ = 0.154 nm), X-ray photoelectron spectroscopy (XPS; ULVAC-PHI Quantera SXM), field emission scanning electron microscopy (FE-SEM; QUANTA 400 F), and high resolution transmission electron microscopy, (TEM; Titan G2 ChemiSTEM Cs Probe) with an energy-dispersive X-ray spectroscopy (EDS) detector.

### Electrochemical performance test

The area of NiCo_2_S_4_ on the flexible stainless steel foil deeped into the electrolyte was kept constant. Electrochemical tests were conducted with a CHI 660E electrochemical workstation in aqueous 1 M KOH electrolyte using a three-electrode cell where the platinum electrode served as the counter electrode, the NiCo_2_S_4_ electrode served as the working electrode, and a standard calomel electrode (SCE) served as the reference electrode.

## Results and Discussion

**Figure 2:**
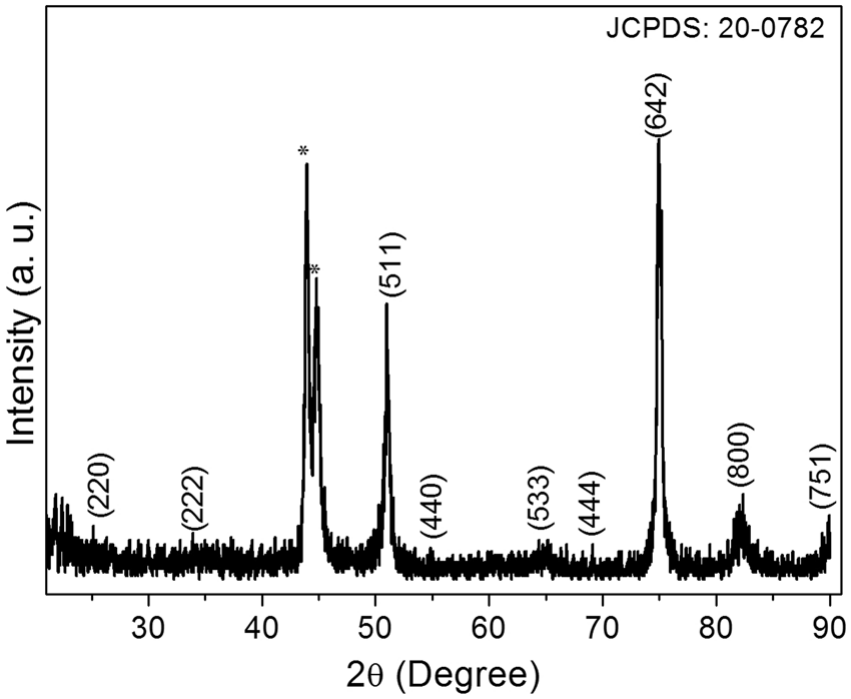
XRD pattern for the NiCo_2_S_4_ sample on the flexible stainless steel foil.

To identify and determine the phase of the NiCo_2_S_4_ sample prepared by the chemical bath deposition method, we first performed XRD measurements, as shown in Fig. 2. Figure 2 shows the XRD pattern of the NiCo_2_S_4_ sample on the flexible stainless steel foil. The diffraction peaks positioned at 25.14°, 33.84°, 51.03°, 54.95°, 66.24°, 69.17°, 74.90°, 82.44°, and 90.74° were indexed to the (220), (222), (511), (440), (533), (444), (642), (800), and (751) planes of the cubic phase of the NiCo_2_S_4_ sample, and all peaks were well matched with the data of the Joint Committee on Powder Diffraction Standards (JCPDS 20-0782). The strong peaks at 43.93° and 44.77° corresponded to the flexible stainless steel foil. The most intense peak was located at 74.90^°^,suggesting the pure/single phase of the NiCo_2_S_4_ material. No other phases such as NiO, CoO, NiCoO_4_, NiS, and CoS were observed in the NiCo_2_S_4_ sample. This result is in agreement with that of a previous study on NiCo_2_S_4_ synthesized by a different method^42–44^. XRD results demonstrated that the chemical bath deposition method is suitable for the preparation of single-phase nanomaterials for applications in energy storage devices. In addition, XRD results are consistent with the results of XPS and FE-SEM.

**Figure 3:**
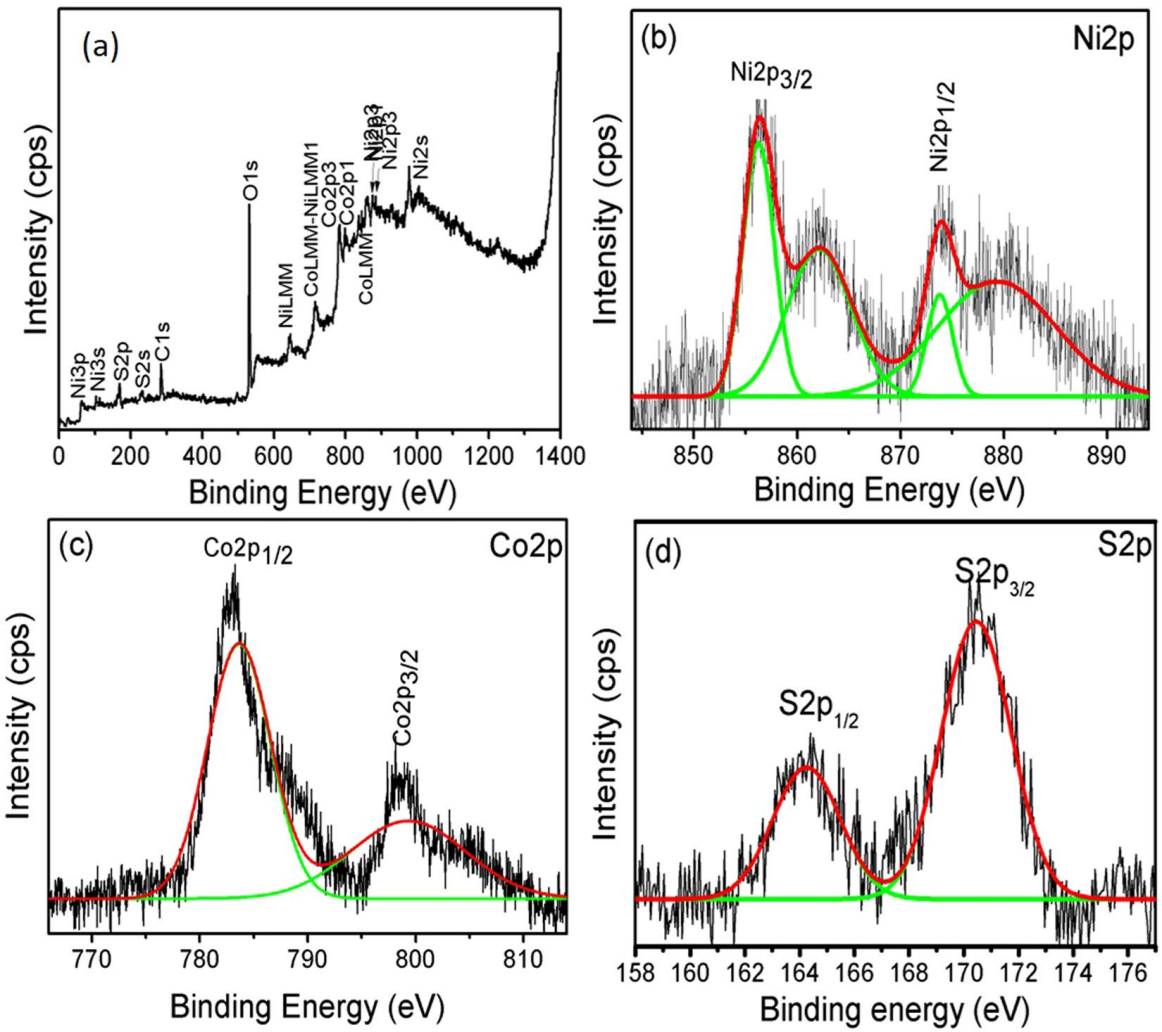
(**a**) XPS survey spectrum of NiCo_2_S_4_ sample, (**b**) high resolution spectrum of Ni2p, (**c**) high resolution spectrum of Co2p, (**d**) high resolution spectrum of S2p.

The chemical state and elemental composition of NiCo_2_S_4_ nanosheets were investigated by XPS measurements, and the corresponding results are shown in Fig. 3(a–d). Figure 3a shows a XPS spectrum of NiCo_2_S_4_ in which peaks are located at 783.24 eV and 798.02 eV corresponded to Co2p, 856.52 eV and 874.03 eV corresponded to Ni2p, and 164.28 eV and 170.57 eV corresponded to S2p, indicating the presence of Ni, Co, and S elements in the NiCo_2_S_4_ sample^45, 46^. In addition, C and O elements were present. The O element was observed because the sample was prepared in double-distilled water. Figure 3(b–d) shows that the high-resolution spectra of the Ni2p, Co2p, and S2p elements can be fitted using a Gaussian fitting method. As shown in Figure S1b, the intensity of all the presented peaks was higher than O1s. Figure 3b shows the high-resolution spectrum of Ni2p. Based on Fig. 3b, the binding energies of 856.52 eV and 874.03 eV were associated with Ni^2+^,whereas those of 862.69 eV and 880.96 eV were associated with Ni^3+^ ^42^. Figure 3c shows the high-resolution spectrum of the Co2p energy level. The binding energies of 783.24 eV and 798.03 eV indicated Co2p, which confirmed that the Co element was present in the NiCo_2_S_4_ sample^42^. Similarly, Fig. 3d shows the high-resolution spectrum of the S2p peak. The binding energies of 164.28 eV and 170.57 eV corresponded to the S2p_1/2_ and S2p_3/2_ energy state of S2p. Figure S1 (a, b) shows the core level of the C1s and O1s elements of the NiCo_2_S_4_ sample^42, 45–48^. These XPS results are comparable with previous XRD analysis results.

**Figure 4:**
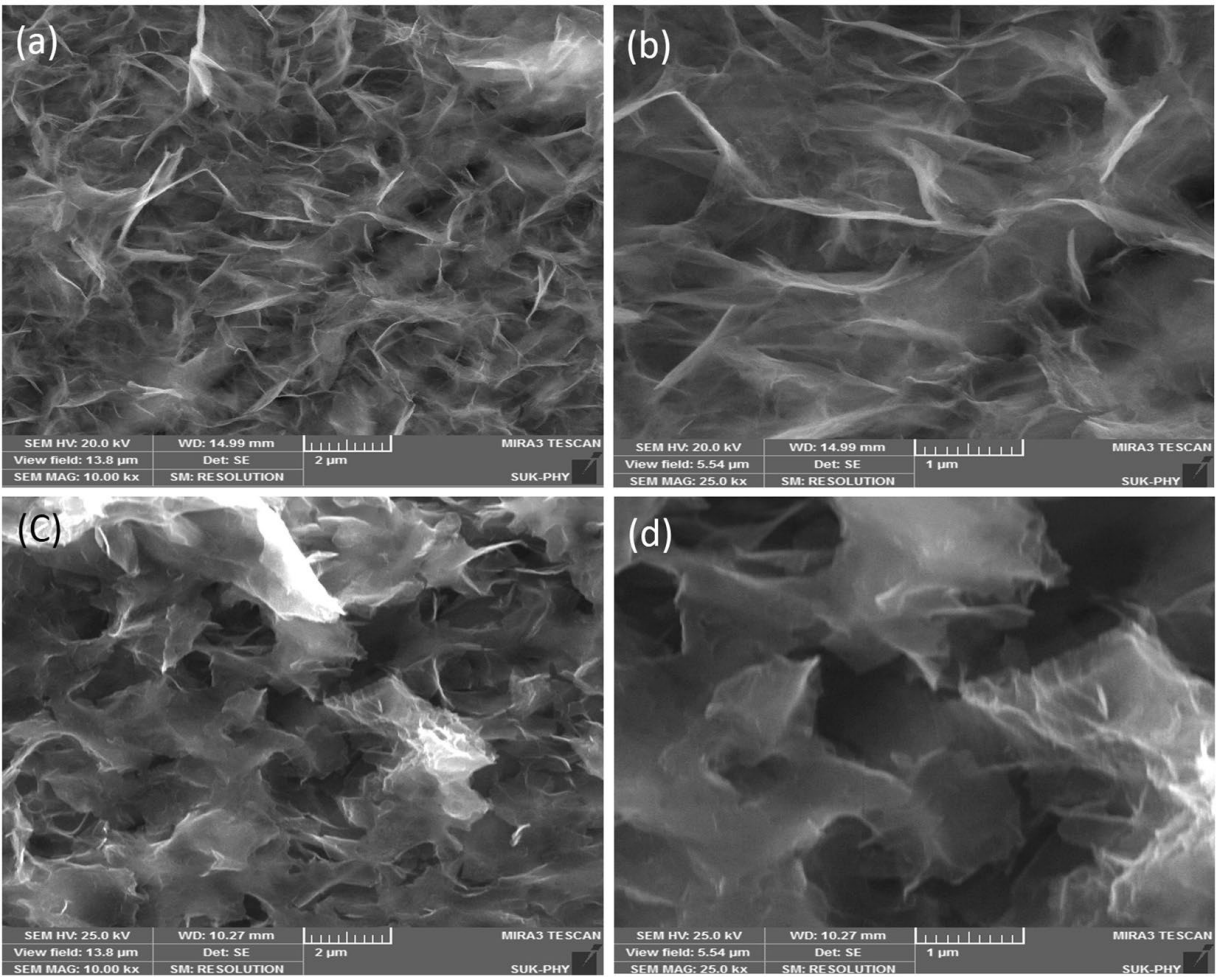
(**a**,**b**) FE-SEM images of NiCo_2_S_4_ sample prepared by chemical bath deposition method, and (**c**,**d**) FE-SEM images of NiCo_2_S_4_ sample after stability testing with different magnifications.

FE-SEM was carried out to obtain further surface information and determine the porosity of the prepared NiCo_2_S_4_ samples before and after stability testing (2000 cycles). Figure 4 (a–d) displays the FE-SEM image of the NiCo_2_S_4_ thin films prepared by the chemical bath deposition method before and after testing the stability of the NiCo_2_S_4_ electrode. Figure 4 (a,b) clearly shows the 3D architecture with the interconnected, highly porous grapheme sheets-like nanosheets distributed on the flexible stainless steel foil^49^. The interconnected uniform 3D nanosheet-like nanostructures of NiCo_2_S_4_ had a thickness of ~30–40 nm. The nanosheets were highly flexible, transparent, and interconnected to each other and had a very low thickness, which demonstrated the large specific surface area of NiCo_2_S_4_^50^. Figure 4(c,d) shows the FE-SEM image of the NiCo_2_S_4_ sample after stability testing. A comparison between Fig. 4(a,b) and Fig. 4(c,d) revealed no change in surface morphology after testing the stability of the sample, thus indicating that the NiCo_2_S_4_ sample is stable. Based on electrochemical measurements, these nanostructures would be beneficial for ion diffusion.

**Figure 5:**
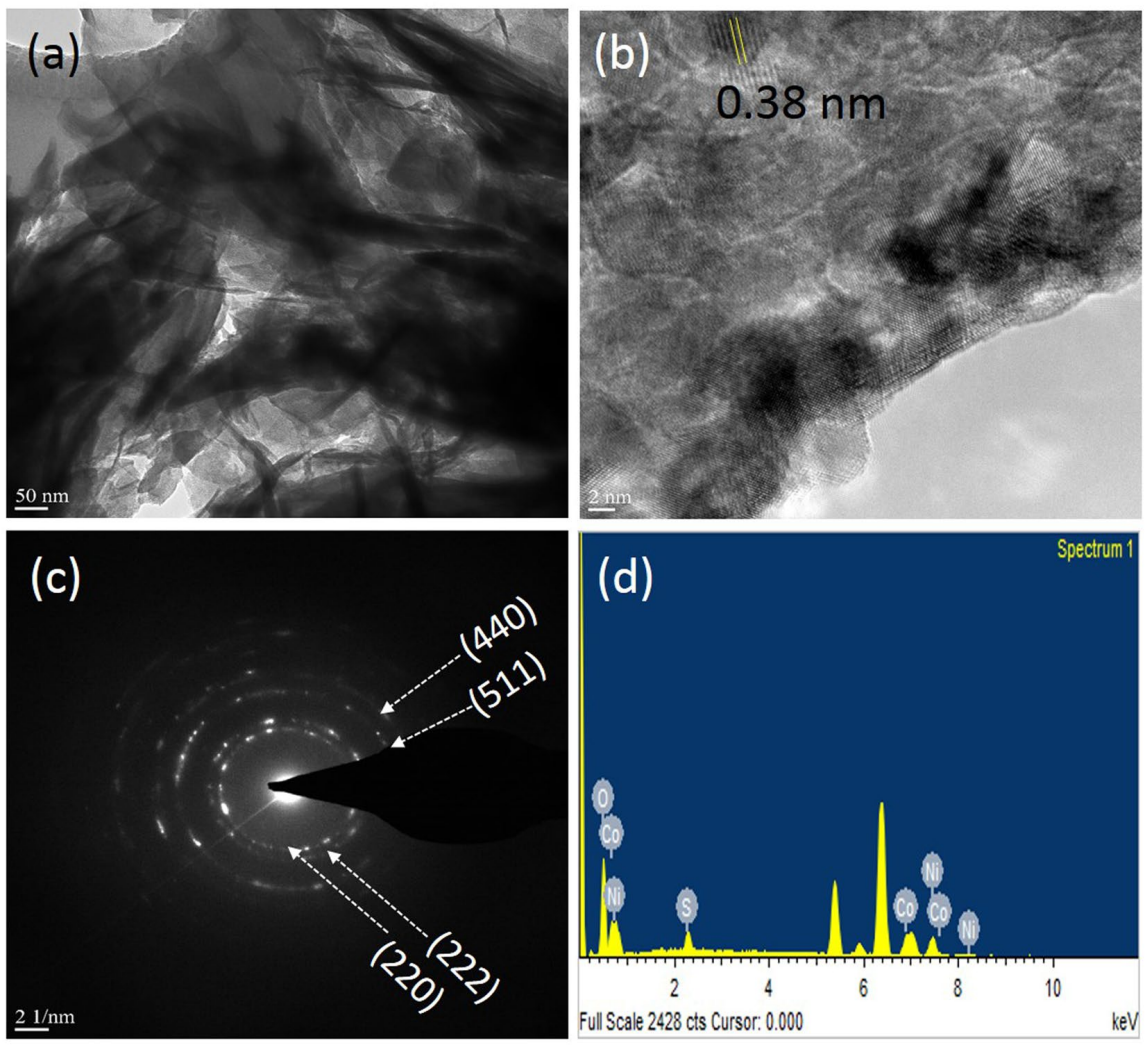
HR-TEM images (**a**,**b**), SAED pattern (**c**), and EDS (**d**), of NiCo_2_S_4_ sample.

**Figure 6:**
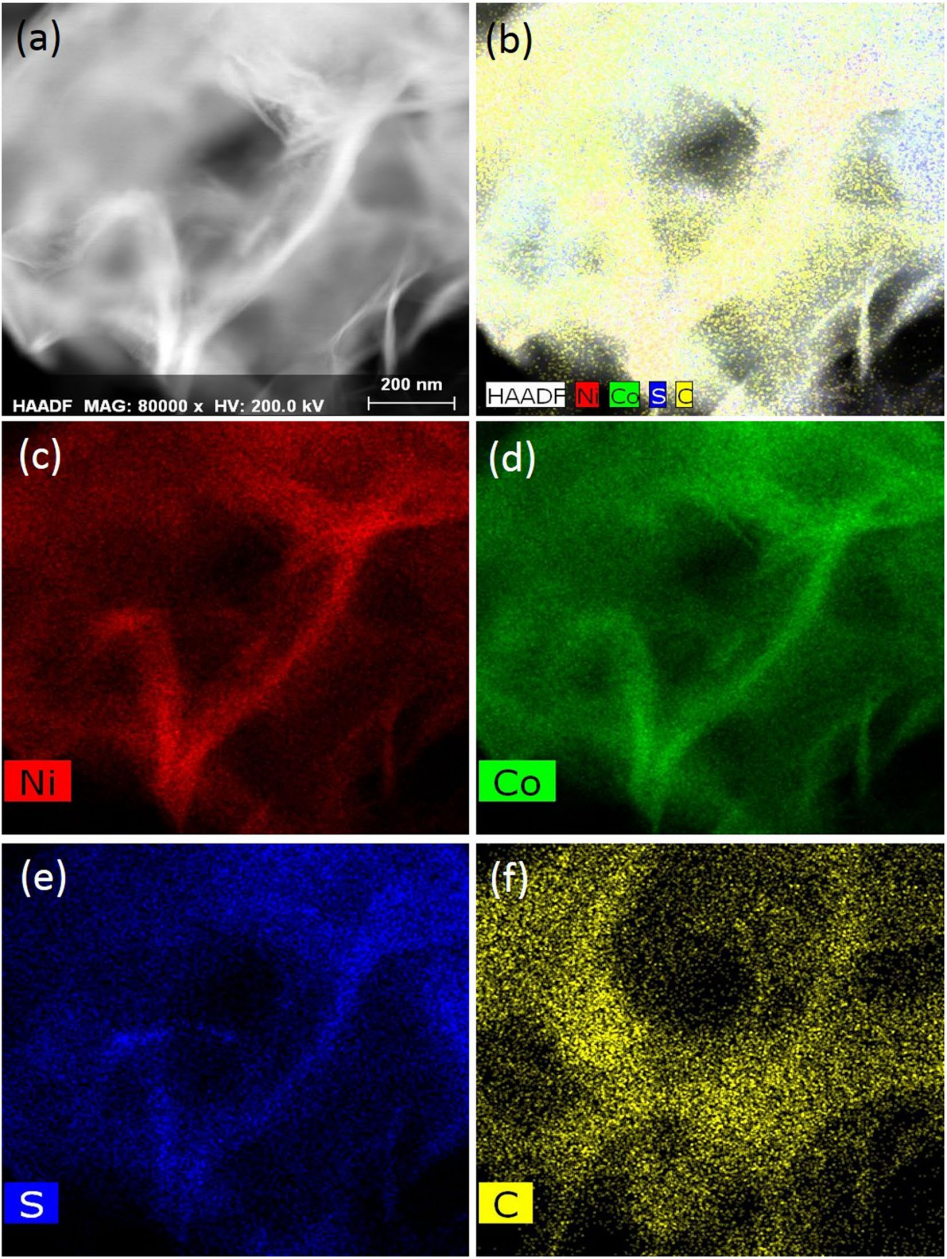
EDS mapping of the NiCo_2_S_4_ sample prepared by chemical bath deposition method.

High-resolution transmission electron microscopy (HR-TEM) was used to perform a detailed assessment of the surface morphology and structural foundation of the NiCo_2_S_4_ sample for supercapacitor applications. Figure 5(a,b) displays typical TEM images of the NiCo_2_S_4_ graphene nanosheets. Figure 5a clearly shows that the length of NiCo_2_S_4_ graphene nanosheets was approximately 90–110 nm, and the graphene nanosheet thickness was approximately 7–10 nm, which indicated the capability for high performance. The interconnected nanosheets result in a larger reactive surface area for supercapacitor applications. Selected area electron diffraction (SAED) pattern was obtained from the HR-TEM image of the NiCo_2_S_4_ sample shown in Fig. 5c. We measured the lattice spacing of the NiCo_2_S_4_ sample in Fig. 5b. The measured lattice spacing was 0.38 nm, which was correlated to the (440) plane of the cubic phase. The graphene-like nanosheets of NiCo_2_S_4_ uniformly covered the flexible stainless steel foil as shown in Fig. 5(a,b), thus supporting the results of FE-SEM. In addition, EDS analysis was conducted to study the elemental composition of Ni, Co, and S. Figure 5d shows the EDS results of the NiCo_2_S_4_ sample, which indicated the presence of the Ni, Co, and S elements in NiCo_2_S_4_. The results are in agreement with those of XPS. Figure 6 shows the EDS mapping of the NiCo_2_S_4_ sample, which revealed that the Ni, Co, and S elements were equally distributed in the sheets.

**Figure 7:**
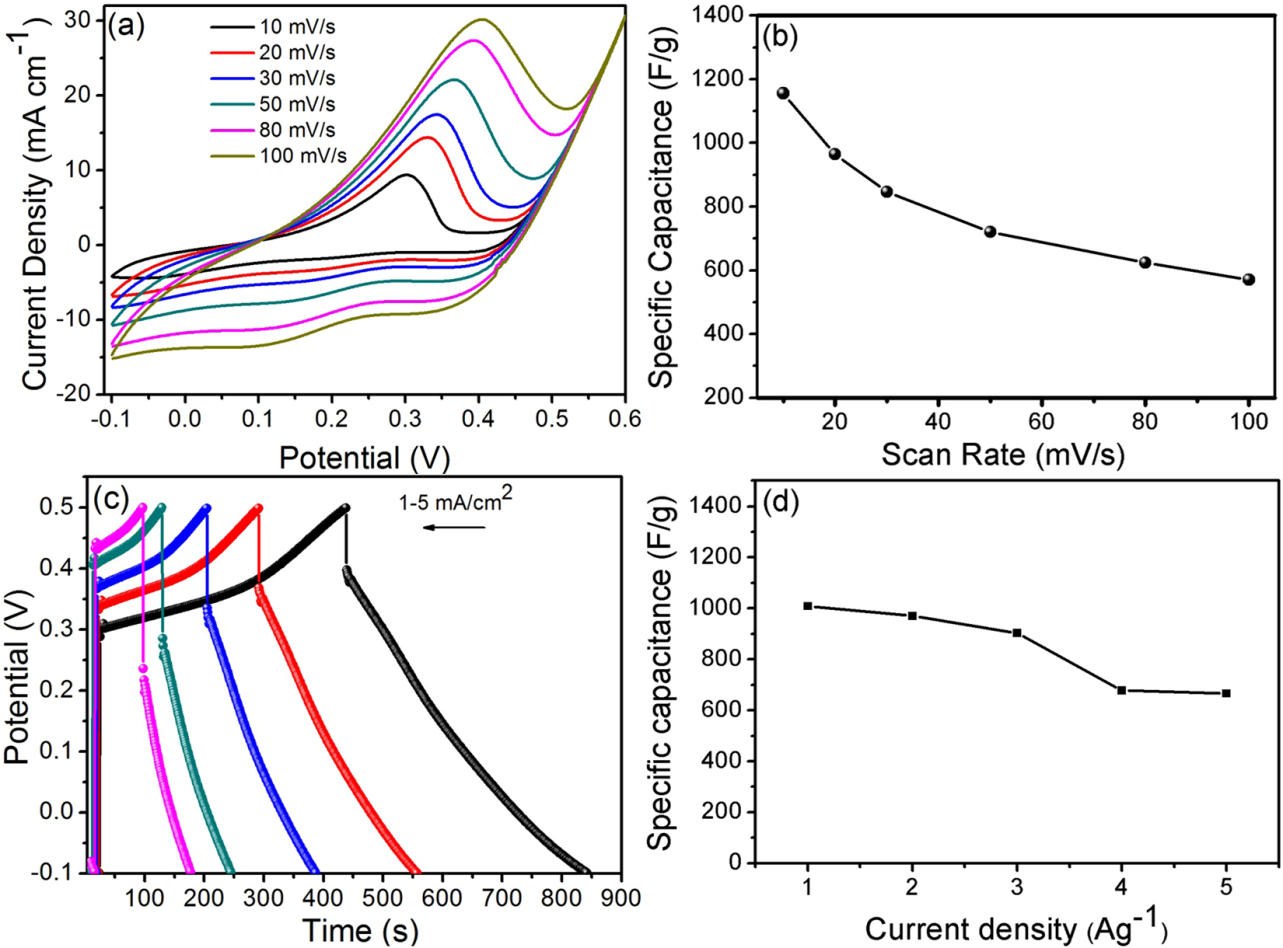
Cyclic voltammetry (CV) curves at different scan rates (**a**), specific capacitance as a function of scan rate (**b**), charging/discharging curves at different current density (**c**), specific capacitance as a function of different current density (**d**), of NiCo_2_S_4_ sample prepared by chemical bath deposition method.

To determine its potential as a candidate for supercapacitor applications, we evaluated the electrochemical performance of the NiCo_2_S_4_ electrode on the flexible stainless steel foil in 1 M KOH electrolyte. Figure 7a displays the typical cyclic voltammetry (CV) curves of the NiCo_2_S_4_ electrode on the flexible stainless steel foil with a potential window of between −0.1 and 0.6 V (Vs SCE) at different scan rates (10–100 mV s^−1^). In the cyclic voltammogram, there was a distinct pair of reduction and oxidation peaks at 0.1 V, 0.35 V, and 0.40 V, which may be attributed to the redox reactions of the NiCo_2_S_4_ electrode in KOH electrolyte^51, 52^. CV demonstrated that the scan rate was increased, and the cathodic current and anodic current densities were increased according to the scan rate (10–100 mV s^−1^), thus indicating the lower resistance of the NiCo_2_S_4_ electrode and fast redox reactions during the electrochemical process. Figure 7b shows the specific capacitance of the NiCo_2_S_4_ electrode at 10–100 mVs^−1^ scan rates in 1 M KOH. A high specific capacitance of 1155 Fg^−1^ was achieved at a scan rate of 10 mV s^−1^, which is comparatively higher than that previously reported in other studies. Zhao *et al* ^53^. have prepared CoNi_2_S_4_ thin films using the hydrothermal method for supercapacitor applications and obtained a maximum specific capacitance of 231.1 mAh g^−1^ at 2 A g^−1^. Pu *et al* ^48^. have synthesized NiCo_2_S_4_ hexagonal nanoplates with a specific capacitance of 437 F g^−1^ at a current rate of 1 A g^−1^ in 3 M KOH aqueous electrolyte.

Figure 7c shows the charge-discharge curves of the NiCo_2_S_4_ electrode at 1–5 mAcm^−1^ current densities. Based on the charge-discharge curves of the NiCo_2_S_4_ electrode, we found that the mirror image of the sample corresponded to the redox reactions during the electrochemical process. In addition, we observed that as the current density increased, the discharge time decreased. The specific capacitances were calculated based on these charge-discharge curves using the following equation:

1 $$C=\frac{I\times {\rm{\Delta }}t}{m\times {\rm{\Delta }}V}$$

where, C (F g^−1^) is the specific capacitance of the electrode, I (A) is the current, Δt (s) is the discharge time, ΔV (V) is the potential window, and m (g) is the mass of active NiCo_2_S_4_ materials. Figure 7d shows the specific capacitance of the NiCo_2_S_4_ electrode with different current densities. Figure 7b and Fig. 7d show similar values of the specific capacitance of the as-synthesized NiCo_2_S_4_ sample. The maximum specific capacitance of the NiCo_2_S_4_ electrode was 1009 F g^−1^ at a current density of 1 mAcm^−1^. The graph shows that as the current densities were increased, the corresponding specific capacitances were decreased. The calculated specific capacitance was higher than that previously reported in the literature^54–56^.

**Figure 8:**
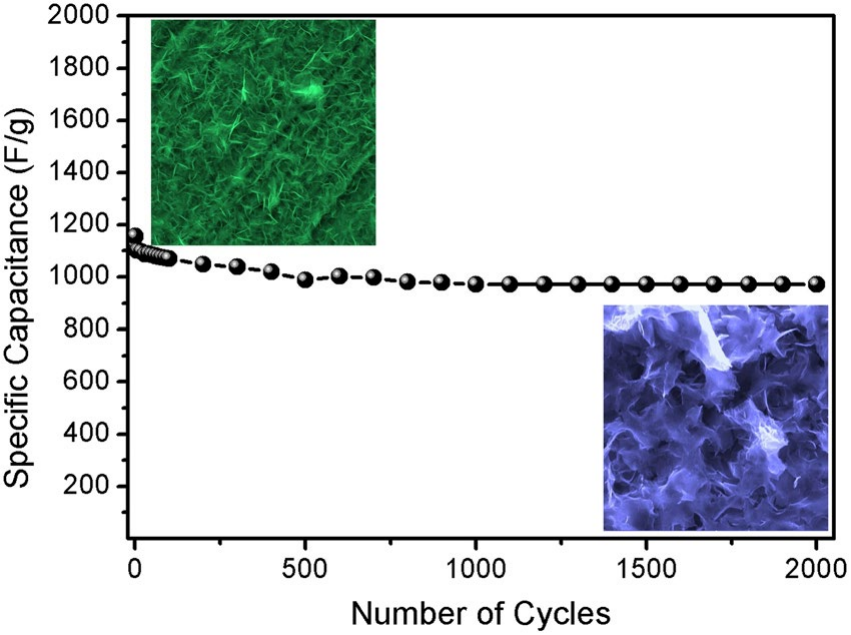
Cycling performance of the NiCo_2_S_4_ electrode at constant scan rate of 100 mV/s.

The most important feature in a supercapacitor is the stability of the materials. To determine the stability of the NiCo_2_S_4_ electrode, the values of specific capacitance with respect to the number of CV cycles at a scan rate of 100 mV s^−1^ were measured^48^, as shown in Figure S2. Figure 8 shows the cycling stability of the NiCo_2_S_4_ electrodes. Figure 8 shows that after the cycling stability testing of the NiCo_2_S_4_ electrodes for 2000 cycles, the specific capacitance was decreased from 1155 to 995 F g^−1^ with a retention of 95%, which was improved in the previously reported NiCo_2_S_4_ samples^48, 56^. This result suggests that the NiCo_2_S_4_ electrode surface is stable during electrochemical reactions; thus, it can be used as a potential material for supercapacitor application.

**Figure 9:**
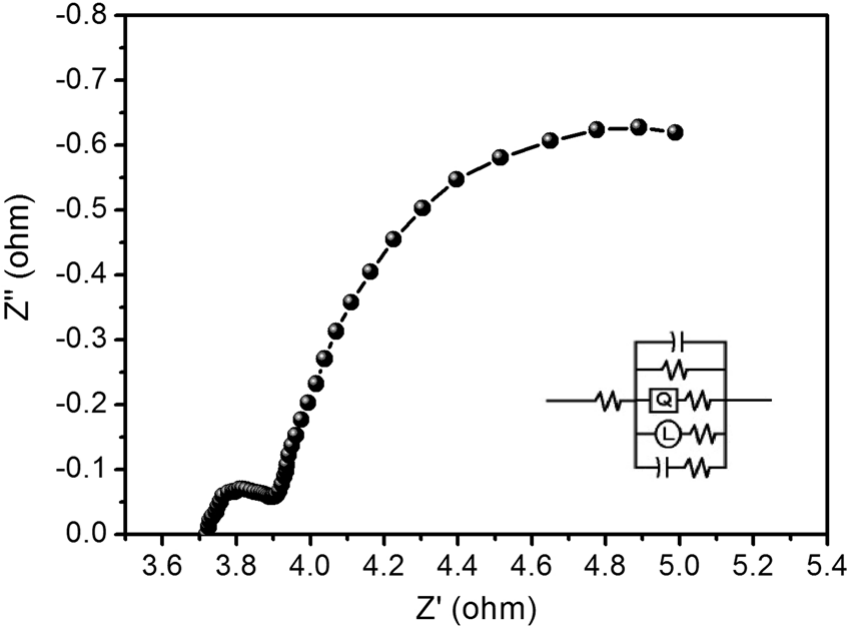
Nyquist plot of NiCo_2_S_4_ sample.

To further understand the mechanism of charge transport and ion diffusion of the NiCo_2_S_4_ electrodes, we performed electrochemical impedance spectroscopy (EIS). EIS measurements were carried out in a frequency range from 100 kHz to 0.01 Hz. Figure 9 shows the Nyquist plots of the NiCo_2_S_4_ electrode synthesized by the chemical bath deposition method. As shown in Fig. 9, semicircles were observed in the high-frequency region, which may be attributed to the resistance of the KOH electrolyte^57^. The linear part of semicircles shows the inclement of the ion diffusion process. The slopes of around 45° in the Nyquist plot indicated the fast ion transfer between the electrode and electrolyte. The values of solution resistance (R_s_) 3.6 Ω and charge transfer resistance (R_ct_) 0.2 Ω are very small. Charge transfer resistance values were low, suggesting that the NiCo_2_S_4_ electrode is suitable for supercapacitor and energy storage applications.

## Conclusion

In this study, novel hierarchical interconnected NiCo_2_S_4_ nanosheets were synthesized on a flexible stainless steel foil using the chemical bath deposition method for high-performance supercapacitors. The nanosheet-like nanostructures of the NiCo_2_S_4_ electrode had a high surface area, specific capacitance of 1155 F g^−1^ at a scan rate 10 mV s^−1^, low solution resistance (3.4 Ω), low charge transfer resistance (0.2 Ω), and good cycling stability after 2000 cycles at 100 mV s^−1^. We proposed that the synthesized NiCo_2_S_4_ nanosheets are promising as electrodes for high-performance energy storage devices.

## Electronic supplementary material

Supplementary Information
