# Supplementary material for: Chemical synthesis of hierarchical NiCo2S4 nanosheets like nanostructure on flexible foil for a high performance supercapacitor
Source: Sci Rep. 2017 Aug 29;7:9764. doi: 10.1038/s41598-017-10218-z (PMC5574986; doi:10.1038/s41598-017-10218-z)
Supplement: Supplementary file 1 — Supplementary Information [file 41598_2017_10218_MOESM1_ESM.pdf]

Chemical synthesis of hierarchical $NiCo_2S_4$ nanosheets like nanostructure on flexible foil for a high performance supercapacitor

D. -Y. Kim[a], G. S. Ghodake[a], N. C. Maile[b], A. A. Kadam[c], Dae Sung Lee[d], V. J. Fulari[b], S. K. Shinde[a*]

[a]Department of Biological and Environmental Science, Dongguk University-Seoul, Biomedical Campus Ilsan, Goyang-si, Gyeonggi-do, 410-773, Korea

[b]Holography and Materials Research Laboratory, Department of Physics, Shivaji University, Kolhapur-416 004, Maharashtra, India

[c]Research Institute of Biotechnology and Medical Converged Science, Dongguk University, Biomedi Campus, Ilsandong-gu, Goyang-si, Gyeonggi-do 10326, Republic of Korea

[d]Department of Environmental Engineering, Kyungpook National University, 80 Daehak-ro, Buk-Gu, Daegu 41566, Republic of Korea

Corresponding author

Prof. Surendra K. Shinde

E-mail: surendrashinde.phy@gmail.com

Tel.: +82-31-961-5122

Fax: +82-31-961-5122

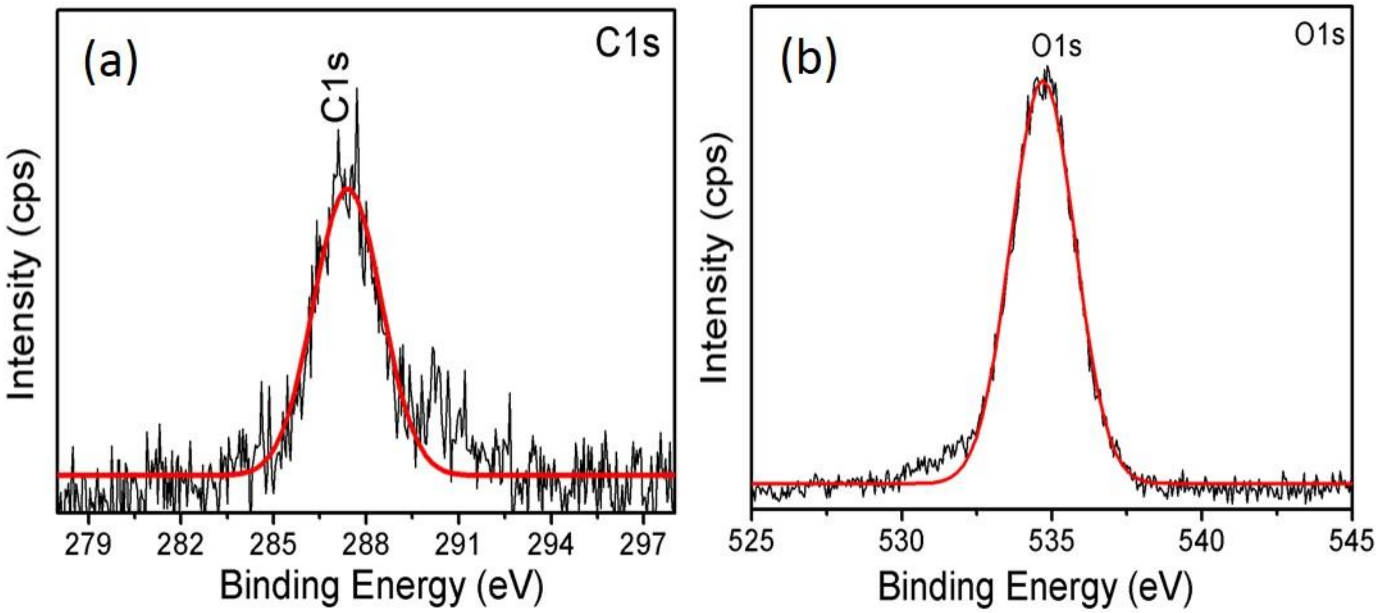

Figure S1 High resolution spectrum of C1s (a), and O1s (b), of $NiCo_2S_4$ sample.

.

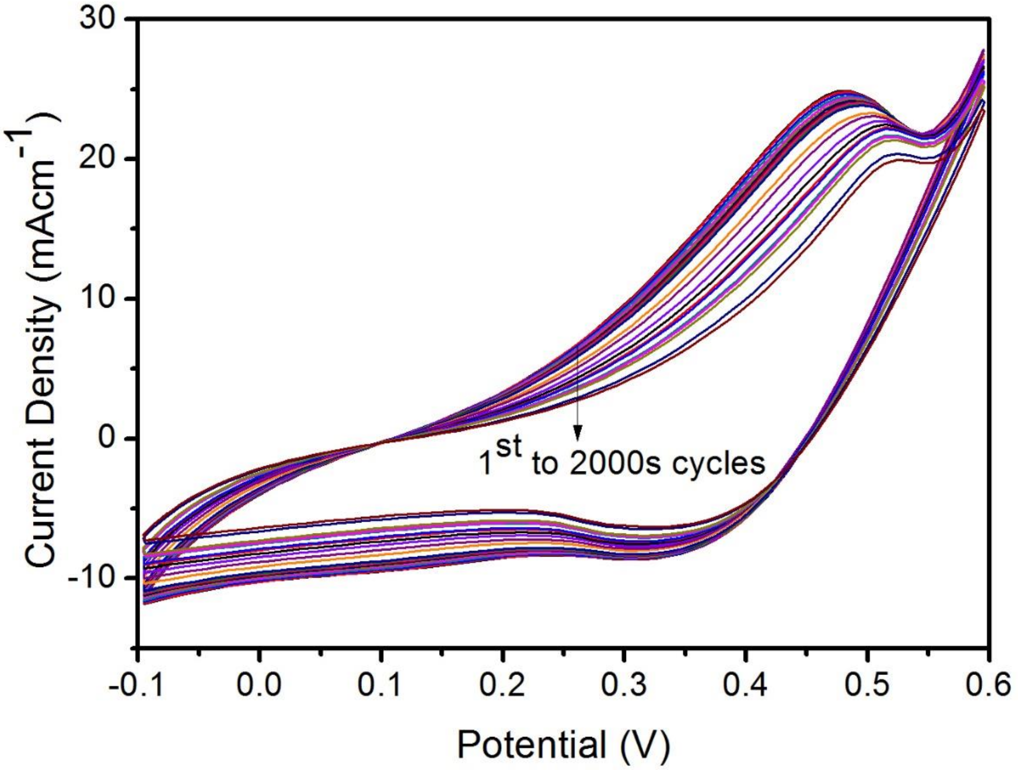

Figure S2 Cyclic voltammetry (CV) curves of $NiCo_2S_4$ electrode at a constant scan rate for the various cycles
